# Phase-tuning Metasurface for Circularly Polarized Broadside Radiation in Broadband

**DOI:** 10.1038/s41598-018-21393-y

**Published:** 2018-02-14

**Authors:** Youfei Zhang, Haogang Wang, Dashuang Liao, Weijie Fu

**Affiliations:** Department of Information Science and Electronic Engineering, Zhejiang University, Hangzhou, 310027 China

## Abstract

Metasurface antennas (MAs) have been proposed as innovative alternatives to conventional bulky configurations for satellite applications because of their low profile, low cost, and high gain. The general method of surface impedance modulation for designing MAs is complicated, and achieving broad operation bandwidth remains a challenge because of its high dispersion response. We propose a novel and easy technique to control cylindrical surface waves radiated by a phase-tuning metasurface. Simultaneously, this technique exhibits a considerably wide working bandwidth. A detailed analysis of the radiation mechanism is discussed. A left-hand circularly polarized (LHCP) antenna and a right-hand circularly polarized (RHCP) antenna that are based on the phase-tuning metasurface are simulated and measured. The measured fractional 3-dB gain bandwidth and gain are higher than 17% and 15.57 dBi, respectively, which are consistent with the simulated results. Moreover, 30% 3-dB axial ratio is achieved for the LHCP and RHCP antennas. To the best knowledge of the authors, it is for the first time to realize a circularly polarized broadband MA by using the phase-tuning mechanism. The approach can be regarded as a new starting point for antenna design, thereby paving the way for the development of broadband and low-profile antennas for future satellite communication.

## Introduction

Metasurfaces, as a type of artificial 2D material that consist of textured scatterers or apertures in the subwavelength scale arranged on a surface, have attracted extensive attention in recent years. The newly structured surfaces exhibit electromagnetic (EM) properties, such as negative refractive index and abrupt phase shift, which are similar to those of 3D metamaterials. In addition, metasurfaces have great potential for EM applications in the microwave and optical frequency regions^1–12^, such as orbital angular momentum generators^3–5^, radar cross section reduction^6–9^, and beam scanning^10–12^, due to their inherent advantages, e.g., ease of fabrication, low loss, and less space occupation. In particular, the metasurface-based linear-to-circular polarization converter has considerable applications in future satellite communications. Various metasurface-based polarization converters^13–21^, such as reconfigurable^13^ and self-complementary^18^ converters, have been proposed recently. However, these polarization converters only function as a cover for the antenna or as a separate device and cannot be integrated into the source.

Recently, the EM manipulation capability of metasurfaces has also drawn the interest of researchers toward antenna design to achieve performance enhancement and size miniaturization^22–27^. The gain of a microstrip-fed slot antenna can be enhanced by using a metasurface as a superstrate^23,24^. The authors of ref.^25^ investigated a wideband filtering antenna with a metasurface. To a certain extent, the aforementioned metasurface-based antennas have improved the compactness of antennas; however, they are still structured by multilayer dielectric. The modulated MA concept was first realized based on the comprehensive analysis of leaky waves on modulated impedance surfaces^26,27^, which demonstrated that a tensor impedance surface could generate circularly polarized radiation with high gain. Modulated MAs exhibit considerable advantages, including lower profile, lower cost, reduced complexity, and lower weight, over conventional MAs. Modulated MAs are constituted by subwavelength patches with various shapes printed on a grounded dielectric substrate. A printed leaky wave antenna was designed and fabricated in 2010 by combining a sinusoidally modulated reactance surface and a slotted parallel-plate waveguide^26^. On the basis of the interaction between a cylindrical surface wave launched by a probe-excited grounded slab and a spiral-shaped modulated inhomogeneous impedance surface, broadside radiation from a bounded TM_0_ surface wave to a circularly polarized beam was achieved^28^. However, an isotropic impedance surface realized via spiral modulation^29^ exhibits the limitation of the cross-polarization level, which is an important parameter of antennas for satellite applications. To overcome this limitation, anisotropic impedance boundary conditions were introduced to a modulated surface, which demonstrated good cross-polarization discrimination performance^30^. Moreover, various possibilities of multibeam and dual-circular polarization antennas have been explored based on anisotropic impedance boundary conditions^31,32^.

However, most current-modulated MAs are based on modulating the impedance of a surface by using a complicated analysis process. Moreover, a large number of units that are significantly smaller than a wavelength are required to structure the impedance surface, which increases manufacturing difficulty. Antenna bandwidth is also limited because of the high dispersion response of subwavelength units. To achieve circularly polarized radiation in a wide frequency band using an easy technique, we propose a phase-tuning mechanism to adjust the phase of currents excited by cylindrical surface waves. In accordance with antenna array theory and Maxwell equations, we can prove that the current-carrying phase ${e}^{\pm i\varnothing }$ that is distributed along the radial direction can radiate RHCP and LHCP beams at the boresight direction. In this study, *ϕ* is the azimuth of the location where the unit is arranged. The phase-tuning function can be realized by varying the scale of the unit. Two circularly polarized MAs are designed, fabricated, and measured. The simulated results show good agreement with the measured results, and the gains of the LHCP and RHCP antennas are both higher than 15.5 dBi. The axial ratio is lower than 0.2 dB at the main lobe direction. Moreover, a wide 3-dB axial ratio and 3-dB gain bandwidth can be obtained. The proposed approach can be regarded as a new starting point for antenna design, thereby paving the way for the development of broadband low-profile antennas for satellite communication.

## Results

### Operation principle

**Figure 1:**
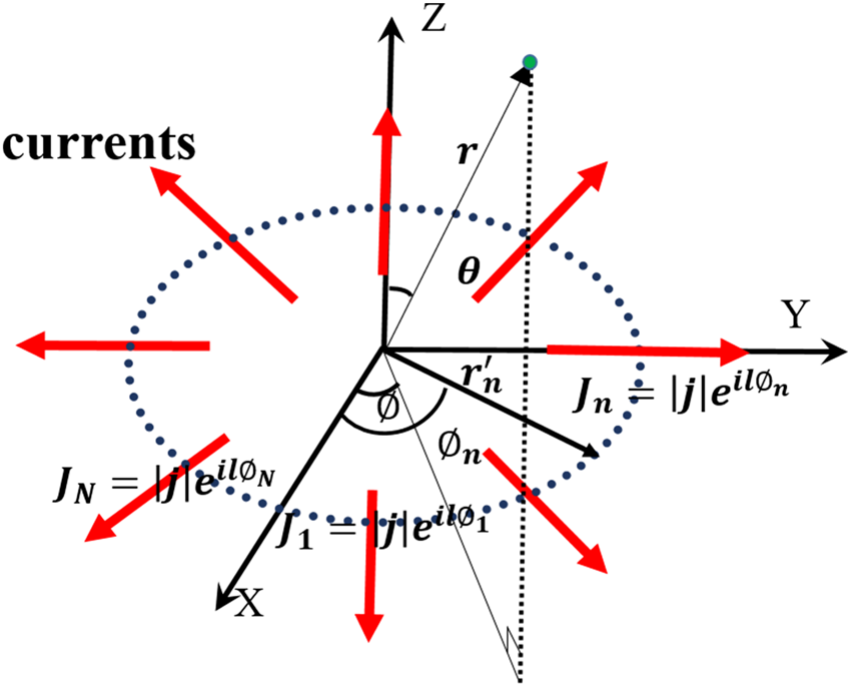
Geometric arrangement of discrete currents on the surface.

To explain the principle of design, we first consider the far field radiated from *N* discrete radial direction currents distributed along the azimuthal direction on the surface at z = 0. To simplify the problem, we disregard the ground and substrate layer and assume that the amplitudes of all the currents are constant. The phase of the *n*th current is selected as ${e}^{\pm il{\varnothing }_{n}}$, where ${\varnothing }_{n}$ is the azimuth of the *n*th current. The geometric arrangement of discrete currents is shown in Fig. 1, where ***r*** is the location vector of the observation point. The time-harmonic factor is selected as ${e}^{-i\omega t}$. To find the magnetic vector ***A***, the expression can be written as follows according to Maxwell’s equations:

1 $${\boldsymbol{A}}=\frac{\mu }{4\pi }{\int }^{}J({\boldsymbol{r}}^{\prime} )\frac{{e}^{ik|{\boldsymbol{r}}-{\boldsymbol{r}}{\boldsymbol{^{\prime} }}|}}{|{\boldsymbol{r}}-{\boldsymbol{r}}^{\prime} |}{\rm{d}}v^{\prime} ,$$

where ***r***′ represents the location vector of the source point and the discrete currents can be expressed as.

2 $${\boldsymbol{J}}({\boldsymbol{r}}^{\prime} )={\boldsymbol{j}}\cdot {e}^{\pm il{\varnothing }_{n}}\cdot \delta ({\boldsymbol{r}}^{\prime} -{{\boldsymbol{r}}}_{n}^{^{\prime} }),$$

where ${\varnothing }_{n}=\frac{2\pi }{N}n$; n = 0, 1, …, *N*−1; ${{{\boldsymbol{r}}}_{n}}^{^{\prime} }=a(\hat{{\boldsymbol{x}}}cos{\varnothing }_{n}+\hat{{\boldsymbol{y}}}sin{\varnothing }_{n})$ denotes the location vector of the *n*th current; $\hat{{\boldsymbol{x}}}$ and $\hat{{\boldsymbol{y}}}$ are the unit vectors; and ${\boldsymbol{j}}=|{\boldsymbol{j}}|(\hat{{\boldsymbol{x}}}cos{\varnothing }_{n}+\hat{{\boldsymbol{y}}}sin{\varnothing }_{n})$ denotes the amplitude and direction of the *n*th current. When Equation (2) is substituted into Equation (1), the solution is reduced to the following expression:

3 $${\boldsymbol{A}}=\frac{\mu }{4\pi }\sum _{n=0}^{N-1}|{\boldsymbol{j}}|(\hat{{\boldsymbol{x}}}cos{\varnothing }_{n}+\hat{{\boldsymbol{y}}}sin{\varnothing }_{n})\cdot {e}^{il{\varnothing }_{n}}\frac{{e}^{ik|{\boldsymbol{r}}-{{{\boldsymbol{r}}}_{{\boldsymbol{n}}}}^{^{\prime} }|}}{|{\boldsymbol{r}}-{{{\boldsymbol{r}}}_{{\boldsymbol{n}}}}^{^{\prime} }|}$$

In the far field, we can approximately make $|{\boldsymbol{r}}-{{{\boldsymbol{r}}}_{{\boldsymbol{n}}}}^{^{\prime} }|\approx r-{\boldsymbol{r}}\cdot {{{\boldsymbol{r}}}_{{\boldsymbol{n}}}}^{^{\prime} }\approx r$, so we rewrite Equation (3) as Equation (4):

4 $${\boldsymbol{A}}=\frac{\mu |{\boldsymbol{j}}|}{4\pi }\frac{{e}^{ikr}}{r}\sum _{n=0}^{N-1}(\hat{{\boldsymbol{x}}}cos{\varnothing }_{n}+\hat{{\boldsymbol{y}}}sin{\varnothing }_{n})\cdot {e}^{il{\varnothing }_{n}}\cdot {e}^{-ik{\boldsymbol{r}}\cdot {{{\boldsymbol{r}}}_{n}}^{^{\prime} }}$$

We can calculate *k****r***·***r***_*n*_′ using following equation:

5 $$\begin{array}{rcl}k{\boldsymbol{r}}\cdot {{\boldsymbol{r}}^{\prime} }_{n} & = & k(\hat{{\boldsymbol{x}}}\,{\sin }\,\theta \,{\cos }\,\varnothing +\hat{{\boldsymbol{y}}}\,{\sin }\,\theta \,{\sin }\,\varnothing +\hat{{\boldsymbol{z}}}\,{\cos }\,\theta )\\  &  & \cdot a(\hat{{\boldsymbol{x}}}\,{\cos }\,{\varnothing }_{n}+\hat{{\boldsymbol{y}}}\,{\sin }\,{\varnothing }_{n})\\  & = & ka\,{\sin }\,\theta \,{\cos }(\varnothing -{\varnothing }_{n})\end{array}$$

Substituting Equation (5) into Equation (4) and using Euler’s formula, we obtain

6 $${\boldsymbol{A}}=\frac{\mu |{\boldsymbol{j}}|}{4\pi }\frac{{e}^{ikr}}{r}\sum _{n=0}^{N-1}[\frac{\hat{{\boldsymbol{x}}}}{2}({e}^{i{\varnothing }_{n}}+{e}^{-i{\varnothing }_{n}})+\frac{\hat{{\boldsymbol{y}}}}{2i}({e}^{i{\varnothing }_{n}}-{e}^{-i{\varnothing }_{n}})]\cdot {e}^{il{\varnothing }_{n}}\cdot {e}^{-ikasin\theta \cos (\varnothing -{\varnothing }_{n})}$$

We can expand ${e}^{-ikasin\theta \cos (\varnothing -{\varnothing }_{n})}$ in Bessel function as

7 $${e}^{-ikasin\theta \cos (\varnothing -{\varnothing }_{n})}=\sum _{m=-\infty }^{+\infty }{i}^{m}{(-1)}^{m}{J}_{m}(kasin\theta ){e}^{im(\varnothing -{\varnothing }_{n})}$$

Substituting Equation (7) into Equation (6) and then the final solution can be reduced to following expression:

8 $$\begin{array}{rcl}{\boldsymbol{A}} & = & \frac{\mu |{\boldsymbol{j}}|}{4\pi }\frac{{e}^{ikr}}{r}[\frac{\hat{{\boldsymbol{x}}}}{2}N{i}^{l+1}{(-1)}^{l+1}{J}_{l+1}(ka\,{\sin }\,\theta ){e}^{i(l+1)\varnothing }\\  &  & +\,\frac{\hat{{\boldsymbol{x}}}}{2}N{i}^{l+1}{(-1)}^{l+1}{J}_{l+1}(ka\,{\sin }\,\theta ){e}^{i(l+1)\varnothing }\\  &  & +\,\frac{\hat{{\boldsymbol{y}}}}{2i}N{i}^{l+1}{(-1)}^{l+1}{J}_{l+1}(ka\,{\sin }\,\theta ){e}^{i(l+1)\varnothing }\\  &  & -\,\frac{\hat{{\boldsymbol{y}}}}{2i}N{i}^{l-1}{(-1)}^{l-1}{J}_{l-1}(ka\,{\sin }\,\theta ){e}^{i(l-1)\varnothing }],\end{array}$$

where ${J}_{l+1}(kasin\theta )$ and ${J}_{l-1}(kasin\theta )$ are the Bessel functions, *l*−1 and *l*+1 represent the order of a Bessel function, and *ϕ* denotes the azimuth of the observation point. From Equation (8), we determine that when *l* is selected as $\pm 1$, which indicates that the phases of the induced currents are ${e}^{\pm i{\varnothing }_{n}}$, a Bessel function with a zero order will obtain the maximum value and other components will be equal to zero at the direction of *θ* = 0. When *l* = 1, Equation (8) can be reduced to

9 $${\boldsymbol{A}}=\frac{\mu |{\boldsymbol{j}}|}{4\pi }\frac{{e}^{ikr}}{r}[\frac{\hat{{\boldsymbol{x}}}}{2}N{J}_{0}(0)-\frac{\hat{{\boldsymbol{y}}}}{2i}N{J}_{0}(0)]=\frac{\mu |{\boldsymbol{j}}|}{4\pi }\frac{{e}^{ikr}}{r}\frac{N{J}_{0}(0)}{2}(\hat{{\boldsymbol{x}}}+i\hat{{\boldsymbol{y}}}).$$

Therefore, the magnetic vector potential ***A*** carries the RHCP characteristic. Correspondingly, when *l* = −1, Equation (8) becomes

10 $${\boldsymbol{A}}=\frac{\mu |{\boldsymbol{j}}|}{4\pi }\frac{{e}^{ikr}}{r}[\frac{\hat{{\boldsymbol{x}}}}{2}N{J}_{0}(0)+\frac{\hat{{\boldsymbol{y}}}}{2i}N{J}_{0}(0)]=\frac{\mu |{\boldsymbol{j}}|}{4\pi }\frac{{e}^{ikr}}{r}\frac{N{J}_{0}(0)}{2}(\hat{{\boldsymbol{x}}}-i\hat{{\boldsymbol{y}}}).$$

Equation (10) shows a LHCP characteristic. Furthermore, we calculate the electric field in the far region as

11 $${\boldsymbol{E}}=i\omega {\boldsymbol{A}}.$$

As shown in Equation (11), the far-field ***E*** in the paraxial direction exhibits the same polarization as ***A***. On the basis of the preceding analysis, a conclusion can be drawn that the induced azimuthally arranged radial currents with phases ${e}^{\pm i{\varnothing }_{n}}$ can generate LHCP and RHCP beams at the boresight direction in the far region. Therefore, if we can devise a unit cell that is capable of tuning the phase of currents to satisfy the aforementioned conditions^33–36^, then we can easily develop antennas with LHCP and RHCP characteristics.

### Unit cell for phase tuning

**Figure 2:**
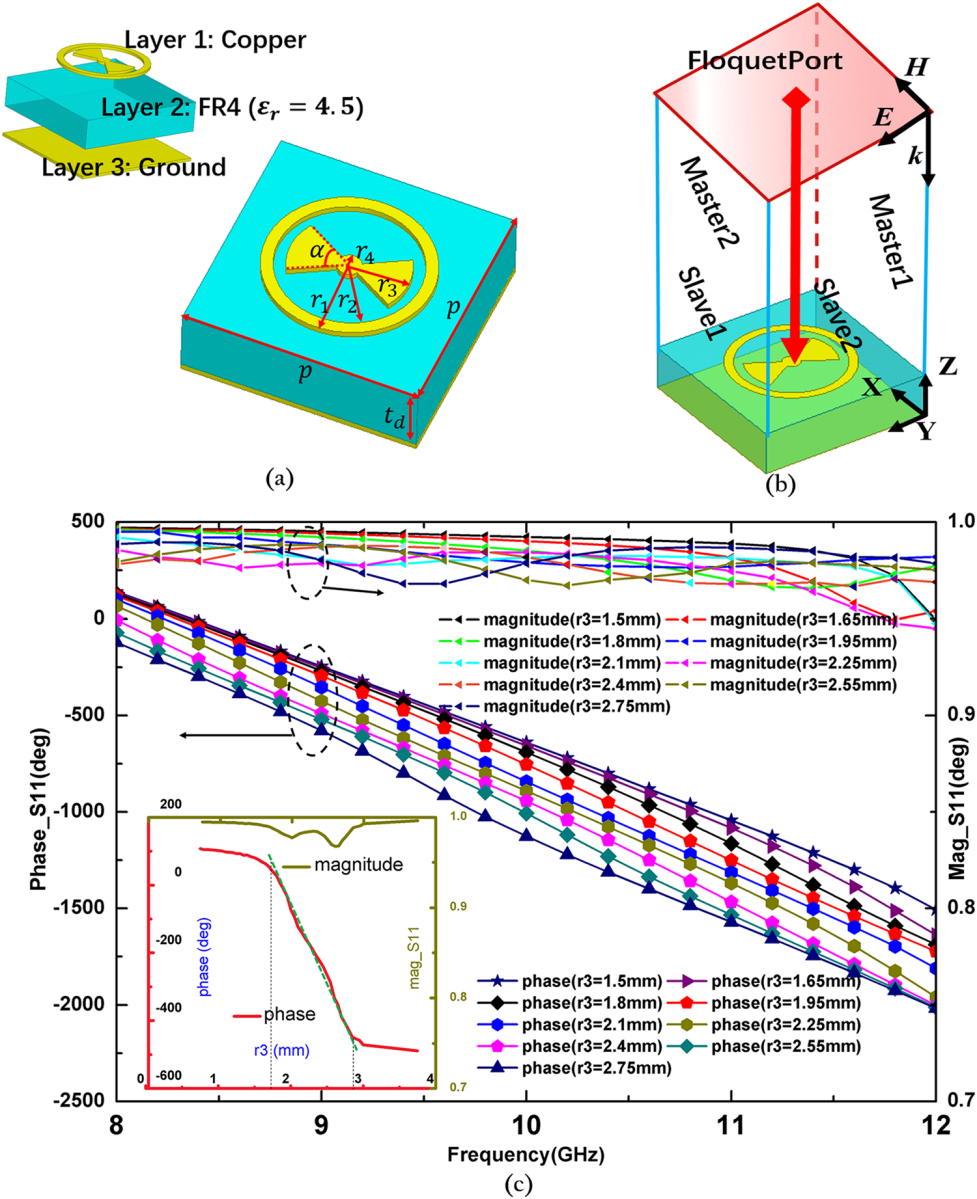
Structure and simulated phase responses of RBU. (**a**) Geometry of RBU, in which the top and bottom layers are modeled as copper and the center layer is a dielectric slab set as FR4 with a relative dielectric constant *ε*_*r*_ = 4.5. The thickness of the substrate is set as td = 3.2 mm, and the period of the unit cell is selected as p = 15 mm. Other sizes of RBU are selected as r_2_ = 1.2r_3_, r1 = 1.4r_3_, r4 = 0.25r_3_, and α = 60°. (**b**) Boundary condition settings for the simulation; master and slave boundary conditions are set at the X and Y directions, and Floquet port is used in the simulation. The incidence electric field is set along the Y direction. (**c**) The phase and magnitude of the simulated reflection coefficients versus frequency with different values of r_3_; inset curves show the phase and magnitude of the reflection coefficient vs. r_3_ at the center frequency of 10 GHz.

If the phase of induced currents needs to be flexibly controlled, then the property of the unit cell is the key. The unit cell must be capable of presenting a phase curve with a sufficiently large range when its physical size varies. For this purpose, the ring-circled bowtie-like unit (RBU) is particularly designed to provide accurate phase compensation. The geometry and main parameters of a unit cell are presented in Fig. 2(a), which consists of a grounded FR4 (*ε*_*r*_ = 4.5) dielectric slab with the RBU printed on the top surface. The RBU comprises a loop and a bowtie-shaped antenna. Each loop provides one resonance; one resonance can generally provide a phase range of over 300°^12^. Therefore, the proposed unit cell can provide a phase range that can be as large as 600°. In addition, the inherent broadband features of a bowtie-shaped antenna guarantee the large operation bandwidth of the proposed device. In this particular design, the thickness of the substrate is set as *t*_*d*_ = 3.2 mm, and the period of the unit cell is selected as p = 15 mm, which is approximately half of a wavelength at the center frequency of 10 GHz. The initial value of *r*_3_ is 5.25 mm. The other sizes of RBU are selected as *r*_2_ = 1.2 *r*_3_, *r*_1_ = 1.4 *r*_3_, *r*_4_ = 0.25 *r*_3_, and α = 60°. To obtain the phase response of the unit cell, a full-wave simulation based on the finite element method (FEM) is implemented. Figure 2(b) shows the simulation setup. The master and slave boundary conditions are set at the X and Y directions. Floquet port is used as the excitation, and the incidence electric field is set along the Y direction, which are consistent with the inner bowtie dipole. The simulated phases and magnitudes of the reflection coefficient S_11_ of the unit cell with different sizes ranging from 8 GHz to 12 GHz are plotted in Fig. 2(c). The inset curves show the phases and magnitudes of the reflection coefficients versus *r*_3_ at the center frequency of 10 GHz, which proves that a phase range of over 600° can be easily achieved by controlling the radius of the bowknot *r*_3_ with negligible disturbances to the magnitude. In addition, the inset curves also indicate that the relation between phase and *r*_3_ is nearly linear when *r*_3_ varies from 1.7 mm to 2.85 mm (shown as a green line), which makes the design process considerably easy. The characteristics of the proposed unit cell make it a good phase tuner for MAs.

### Simulation and measurement

**Figure 3:**
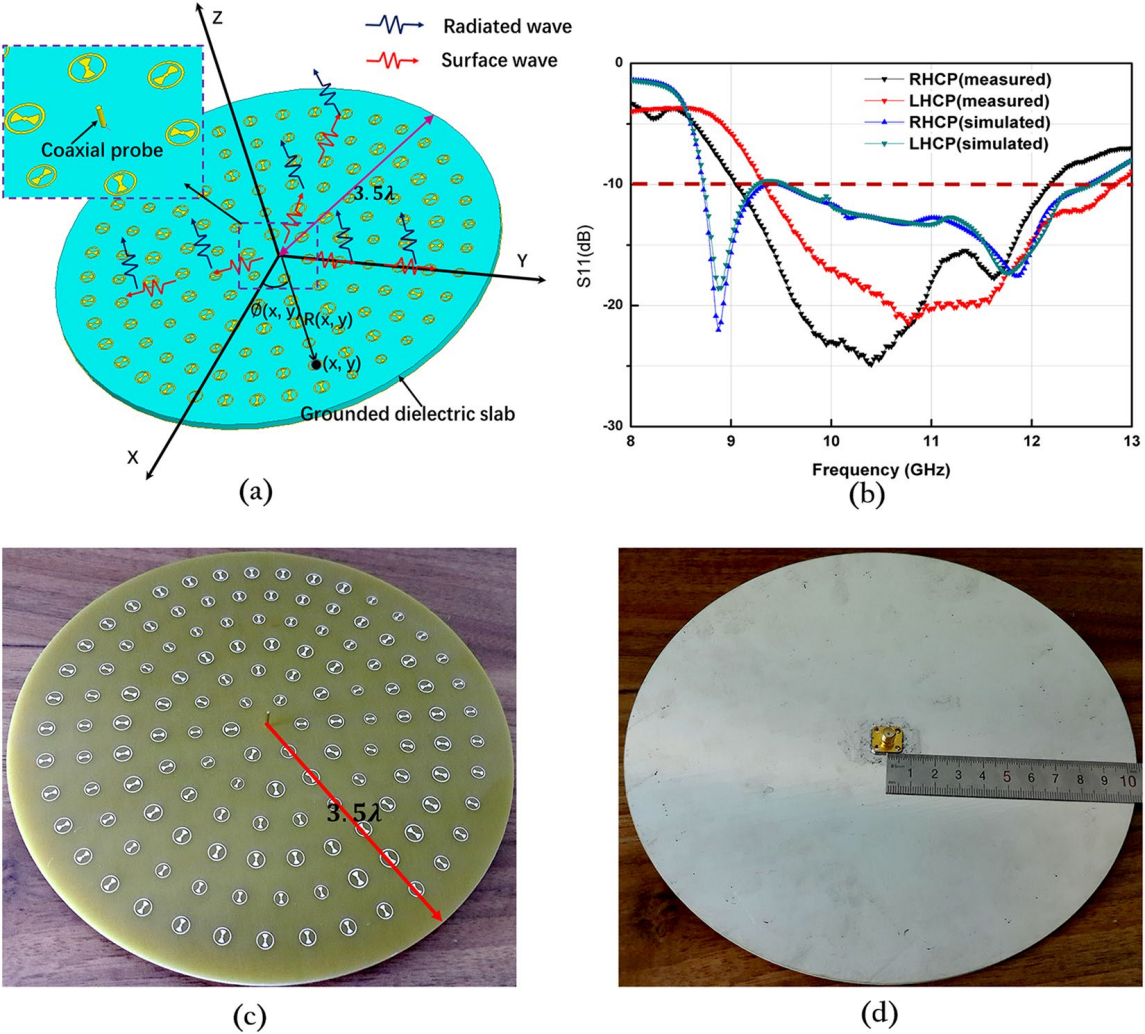
(**a**) Configuration of the proposed MA. Red zigzag lines represent the surface wave excited by coaxial probe, and black zigzag lines represent electromagnetic wave radiated by RBUs. *ϕ* (x, y) is the azimuthal angle of position (x, y). (**b**) Front view of the fabricated MA. (**c**) Rear view of the fabricated MA. (**d**) Simulated and measured reflection coefficients of LHCP and RHCP MAs.

**Figure 4:**
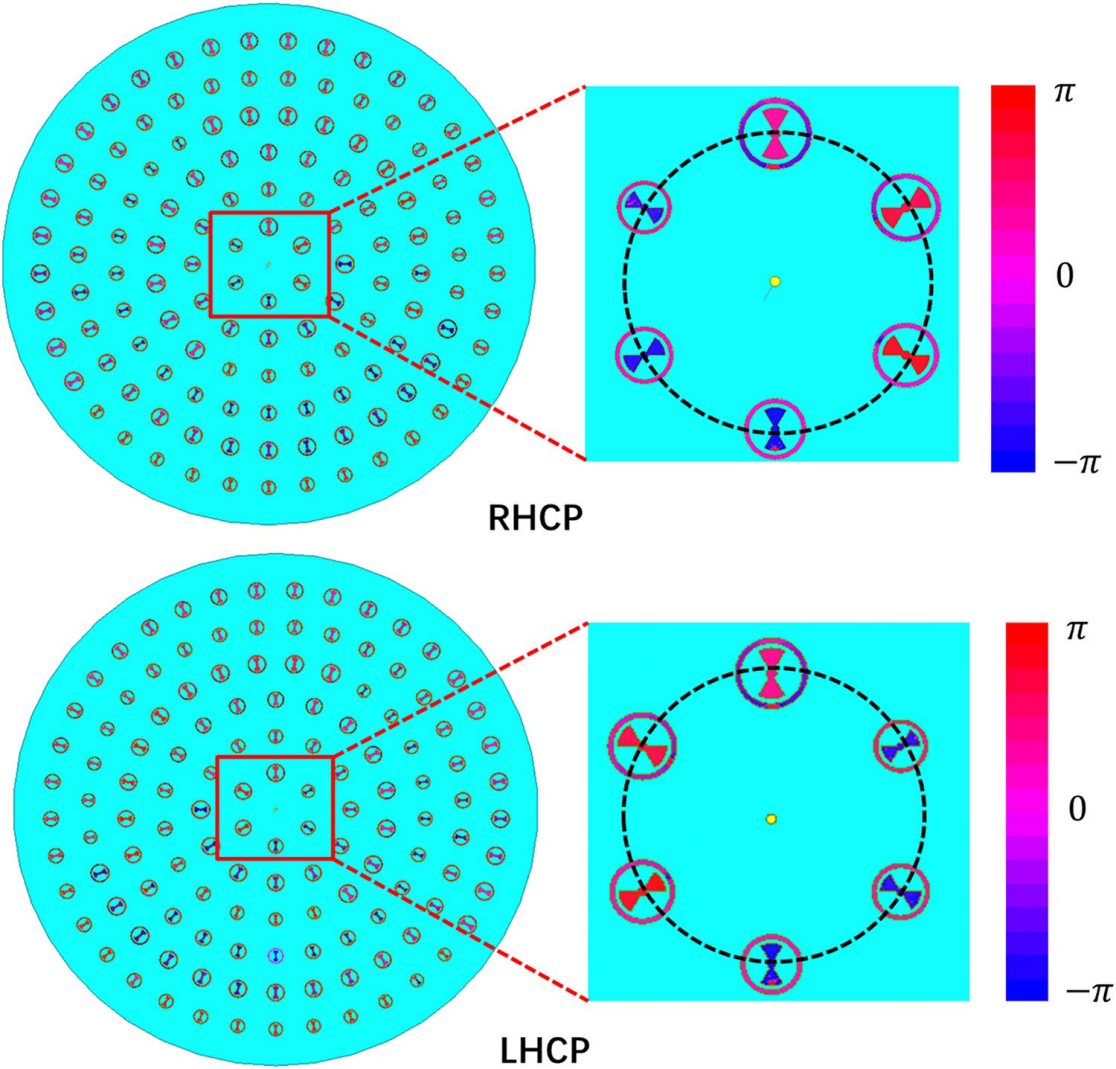
Phases of surface currents of the (**a**) LHCP and (**b**) RHCP MAs.

**Figure 5:**
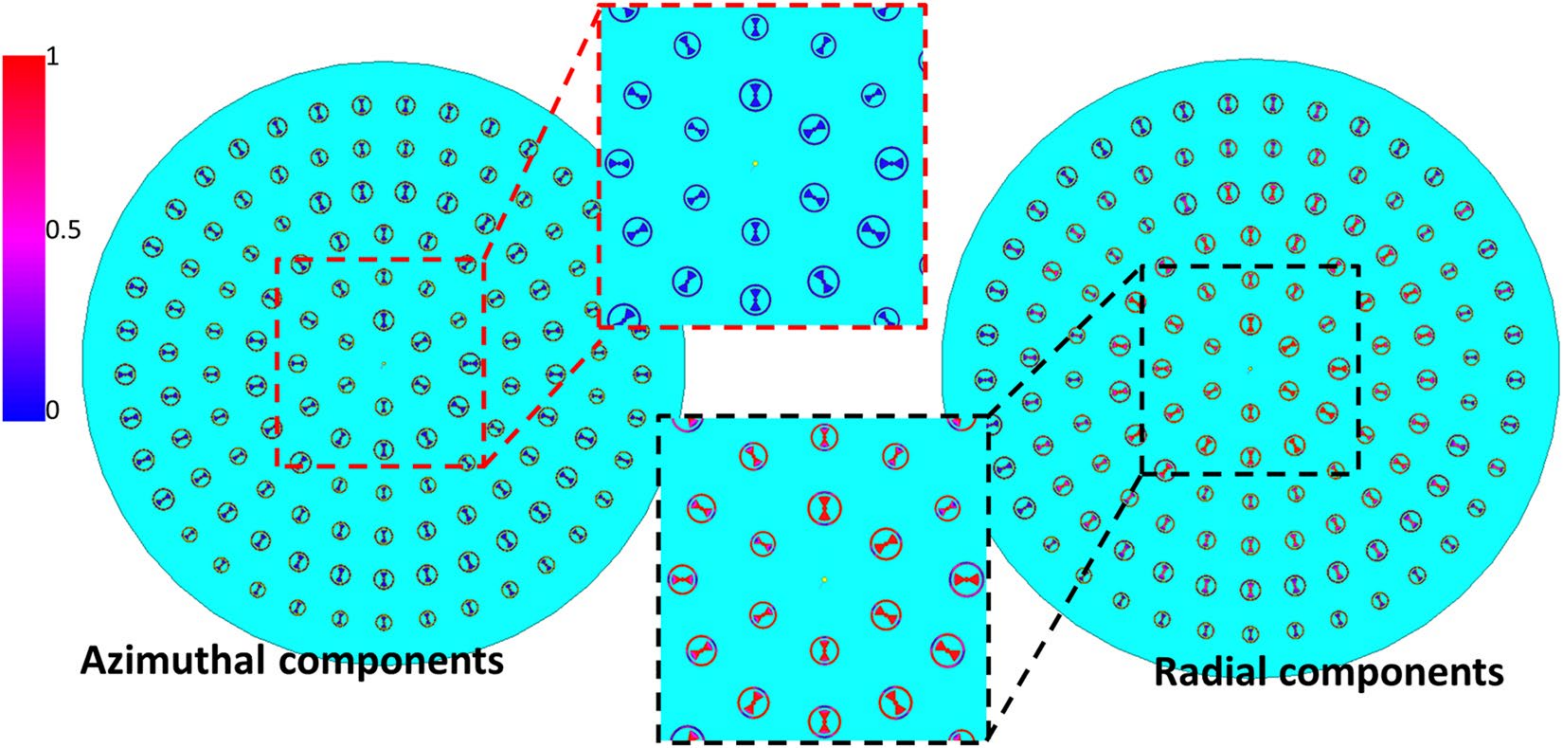
The magnitude of the radial and azimuthal component of surface currents of the RHCP MA.

Figure 3(a) depicts a 3D view of the proposed MA, which is structured by assigning RBUs on a grounded dielectric slab. To obtain radial direction currents, RBUs are arranged with the inner bowtie along the radial directions. The phase-tuning factor *ϕ*_c_(*x*, *y*) must be introduced to make the phases of the induced currents at position (*x, y*) equal to ${e}^{\pm i\varnothing (x,y)}$, where *ϕ* (*x*, *y*) is the azimuthal angle of position (*x, y*). *ϕ*_c_(*x*, *y*) should be obtained as

12 $${\varnothing }_{c}(x,y)=\pm i\varnothing (x,\,y)-{{\boldsymbol{k}}}_{s}\cdot {\boldsymbol{R}}(x,y)+2n\pi ,$$

where ***k***_***s***_ is the surface wave vector, ***R***(*x*, *y*) denotes the position vector of point (*x*, *y*), and ${{\boldsymbol{k}}}_{s}\cdot {\boldsymbol{R}}(x,y)$ represents the propagating phase delay of surface waves from the center to position (*x*, *y*). In addition, integer *n* is selected to ensure that *ϕ*_c_(*x*, *x*) will be between 0 and 2 π. Figure 4 shows the phase distributions of surface currents of units. From Fig. 4, whether for LHCP or RHCP MA, a phase change from 0 to 2π can be observed, and it is consistent with the expression of *ϕ* (*x*, *y*). In addition, in order to verify the fact that the induced surface currents is mainly in radial direction, the magnitudes of radial component and azimuthal component of surface currents of the RHCP MA as an example are given in Fig. 5. It can be seen that the magnitudes of the radial components are much larger than the magnitudes of azimuthal components, and the magnitudes of the azimuthal components are almost zeros.

**Figure 6:**
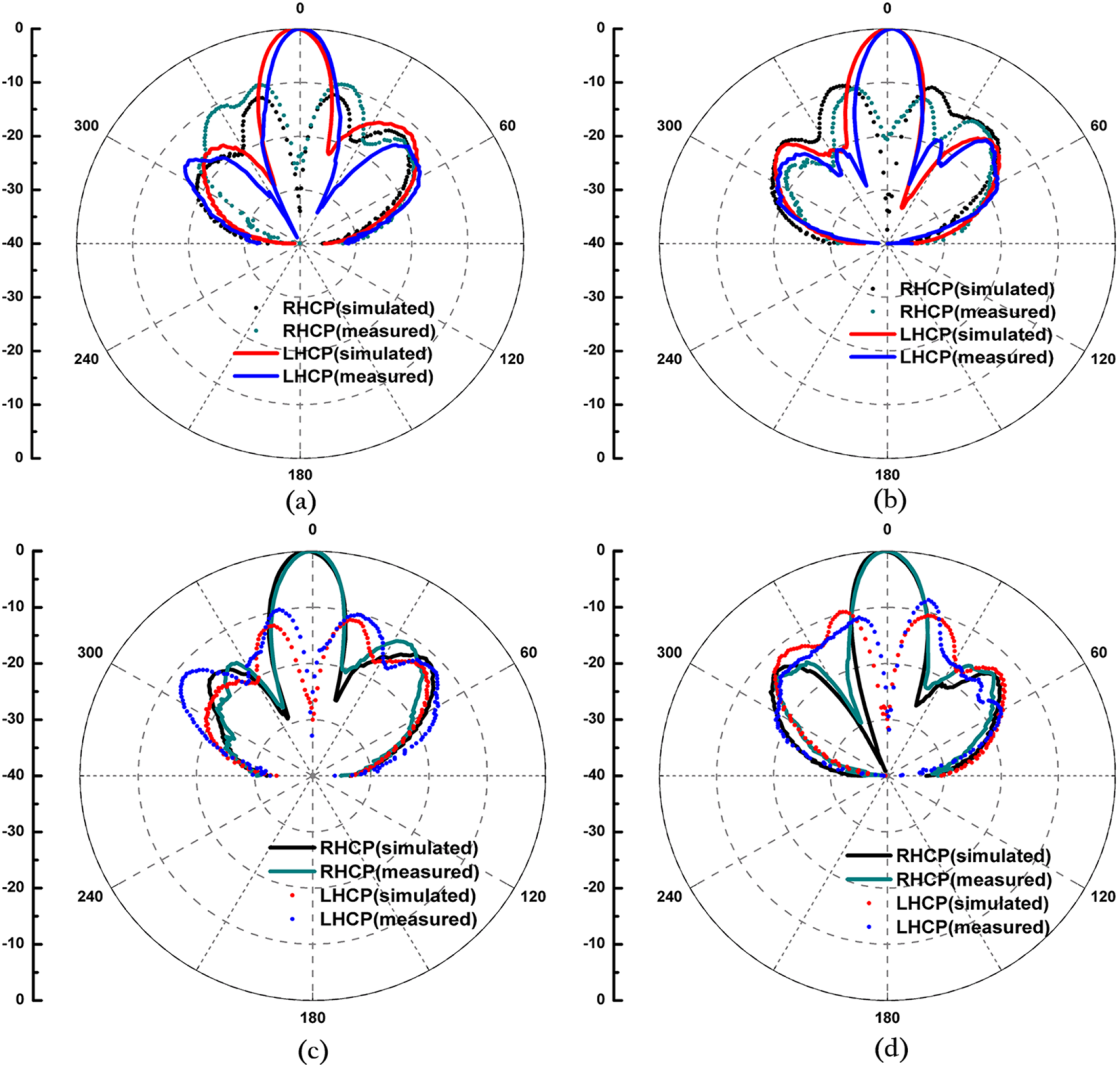
Simulated and measured radiation patterns of the LHCP antenna in the (**a**) X–Z and (**b**) Y–Z planes and the RHCP antenna in the (**c**) X–Z and (**d**) Y–Z planes at 10 GHz.

**Figure 7:**
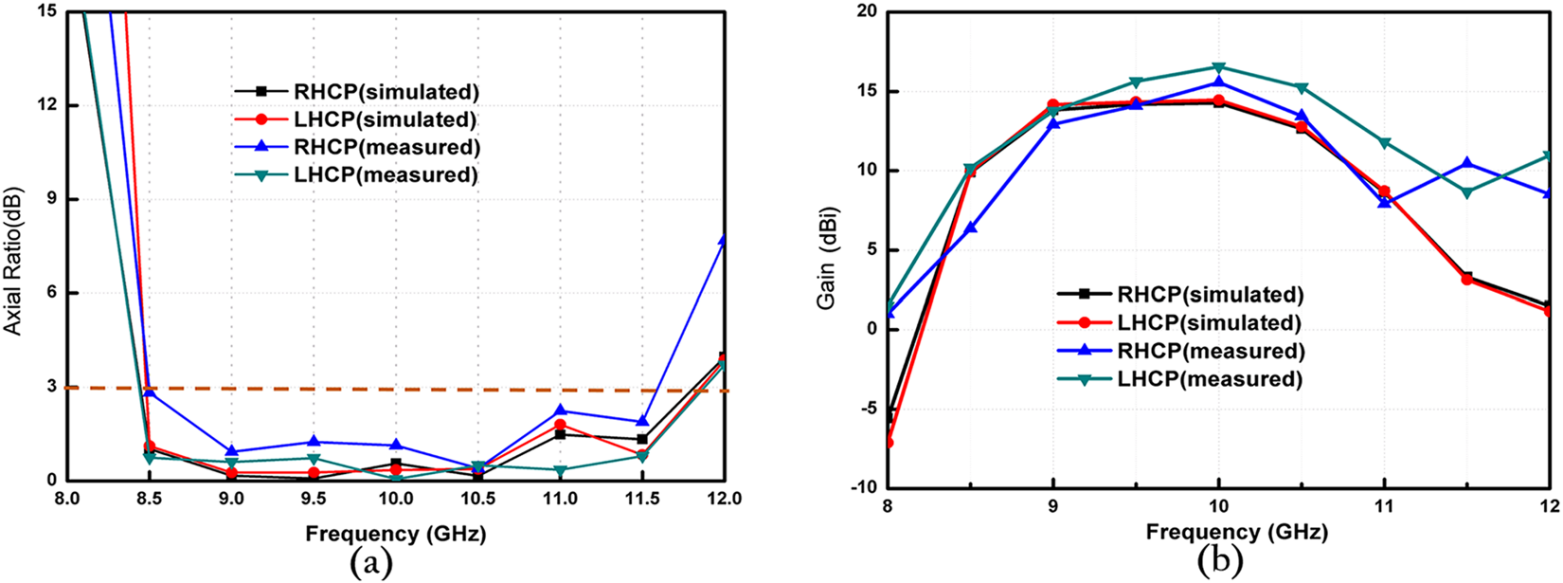
Simulated and measured (**a**) axial ratios and (**b**) gains of the LHCP and RHCP MAs.

**Figure 8:**
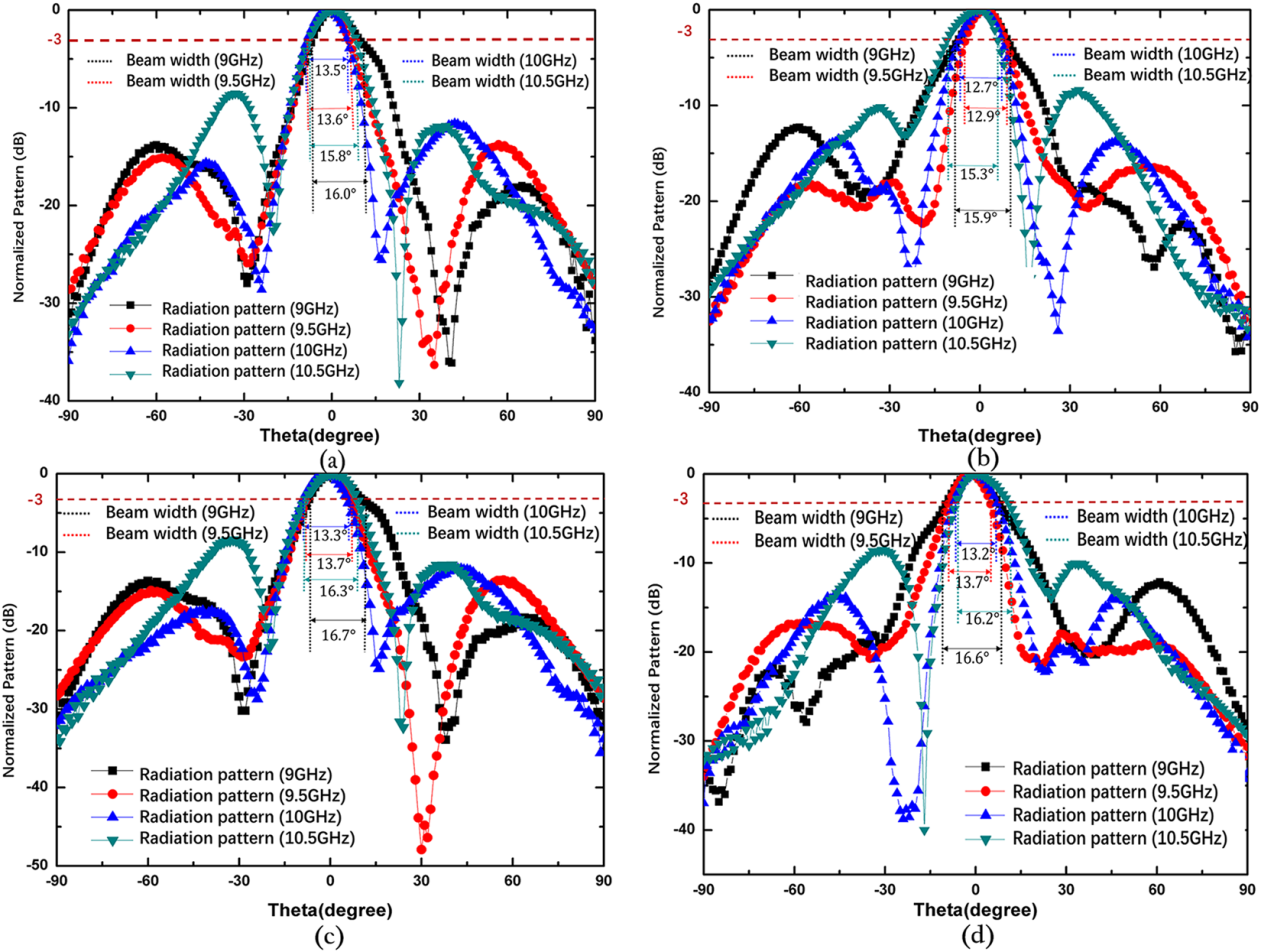
Simulated normalized radiation patterns and beam widths of the proposed MAs at four different frequencies of 9 GHz, 9.5 GHz, 10 GHz and 10.5 GHz. (**a**) Radiation patterns of the LHCP antenna in the X–Z planes. (**b**) Radiation patterns of the LHCP antenna in the Y–Z planes. (**c**) Radiation patterns of the RHCP antenna in the X–Z planes. (**d**) Radiation patterns of the RHCP antenna in the Y–Z planes.

**Figure 9:**
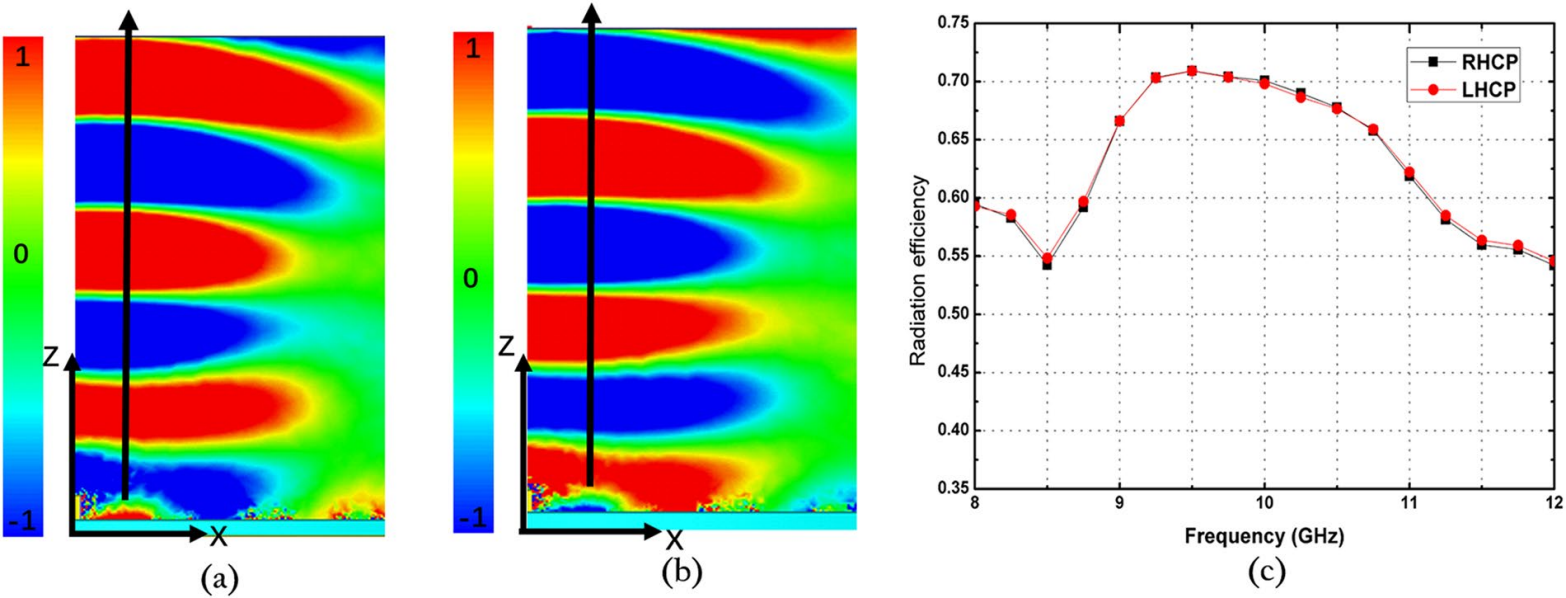
Near-field distribution in the X–Z plane at 10 GHz and radiation efficiency of the antenna. (**a**) *E*_*y*_ excited by the LHCP antenna, (**b**) *E*_*y*_ excited by the RHCP antenna, and (**c**) radiation efficiency of the antennas at different frequencies.

To confirm the proposed antenna, we fabricate and measure the LHCP and RHCP MAs with a radius of 3.5 *λ*, where *λ* is 30 mm and the thickness of the grounded slab is 3.2 mm. Figure 3(c) and (d) show the photograph of the fabricated LHCP antenna. Figure 3(b) presents the simulated and measured reflection coefficients of the LHCP and RHCP MAs, in which the difference between the simulation and the measurement originates from the length error of the probe. In the simulation setup, the probe length is set as 0.25 *λ*. In the experiments, however, we gradually cut a long probe to achieve better impedance matching, and thus, decrease the influences of material and fabrication errors on the results. In such process, ensuring that the simulation result is completely identical to the experiment result is difficult. Nevertheless, S_11_ can remain lower than −10 dB within a wide frequency band. From the measured results, the relative impedance bandwidths of the LHCP and RHCP MAs achieve approximately 31% (9.075–12.175 GHz) and 34.75% (9.35–12.825 GHz), respectively. To validate the radiation characteristics of the proposed antenna, the radiation patterns of the co-polar and cross-polar components of the LHCP and RHCP MAs are simulated and measured at 10 GHz. Figure 6 shows the simulated and measured normalized radiation patterns at two orthogonal cuts. A comparison of the results shows that the measured results exhibit satisfactory agreement with the simulated results. Figure 6 clearly illustrates that the LHCP and RHCP antennas can obtain maximal radiation at the broadside direction and the cross-polar levels are lower than −10 dB for both cuts. In particular, cross-polar components can be less than −35 dB at the main lobe directions. To better recognize the performance of the antenna, the axial ratios and gains of the two circular polarized antennas are also simulated. The measured results are plotted in Fig. 7(a) and (b). These figures show that the measured axial ratios and gains agree well with the simulation. The slight discrepancy is mainly caused by fabrication and measurement errors. As shown in Fig. 7(a), the axial ratios of the RHCP and LHCP antennas are lower than 3 dB above 3 GHz (30%) bandwidth of the measured results, and the value reaches 0.058 dB and 0.149 dB at the working frequency of 10 GHz, thereby confirming the good polarization characteristics of the proposed MAs. Figure 7(b) shows that the gain can approach 15.57 dBi for the RHCP antenna and 16.56 dBi for the LHCP antenna, and the 3-dB gain bandwidths approach 17% (8.9–10.6 GHz) for the RHCP antenna and 22% (9–11.2 GHz) for the LHCP antenna. Figure 8 shows the simulated normalized radiation of the LHCP and RHCP MAs at 9, 9.5, 10, and 10.5 GHz in the X–Z and Y–Z planes. Correspondingly, the simulated main beam is pointed toward −2°, −0.7°, 0, and 1°. In addition, the beam widths of the LHCP and RHCP antennas increase when the working frequency deviates from the designed value of 10 GHz regardless of whether in the X–Z or Y–Z plane. As mentioned earlier, the phase responses of the RBU at 10 GHz are selected to structure the phase-tuning metasurface. However, Fig. 2(c) shows that the relations between phase and *r*_3_ are not completely identical at different frequencies. Therefore, increasing beam deviation and beam width occurs if the working frequencies deviate from the designated center frequency of 10 GHz. Moreover, Fig. 9(a) and (b) present the distributions of *E*_*y*_ (the *y*-axis components of the electric field) as an example of near-field properties to improve understanding. From the figures, the near fields mainly propagate along the boresight direction, and the electric field at the edge of an antenna is extremely weak, which can be explained by leaky wave theory. Cylindrical surface waves launched by a probe are gradually radiated by the RBUs assigned on a slab when they propagate from the center to the edge. Thus, the electric fields will be weak as they move closer to the edge of the antenna. Figure 9(c) plots the simulated radiation efficiency of the two proposed antennas from 8 GHz to 12 GHz, and the curves illustrates that radiation efficiency at 10 GHz can be approximately 70%. Table 1 presents the key performance of several recently reported MAs for comparison. As illustrated, the phase-tuning MA proposed in this work has a considerably larger 3-dB gain bandwidth than those in other works.

**Table 1:** Comparison of the measured performance of recently reported MAs.

Works	Physical area of antenna(λ^2^)	Maximum gain(dBi)	Fractional 3-dB gain bandwidth (%)
Ref.^13^	235.62	25.5	5.75
Ref.^20^	18.5	13	8.33
Ref.^21^	162.9	28.3	2.4
Ref.^25^	69.4	22	10
Ref.^27^	201.1	25	1.5
This work	38.5	16.56	21.68

**Figure 10:**
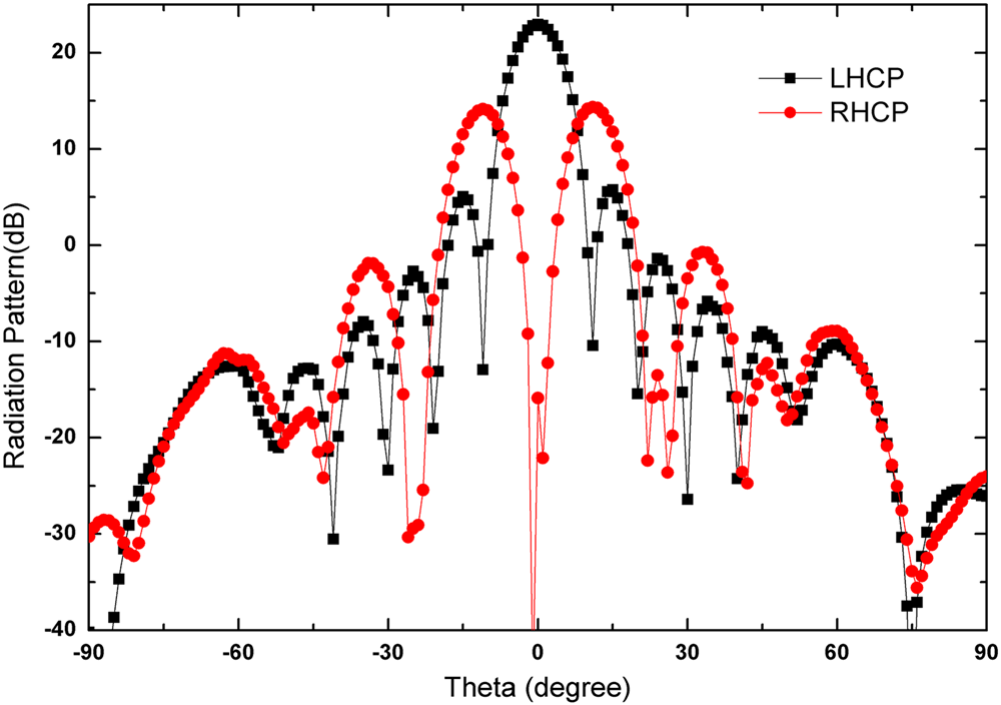
Radiation patterns of the Hertzian dipole array on a grounded slab for the LHCP radiation.

From another perspective, the top layer of each RBU can also be regarded as a small dipole. Then, the whole MA will be a phased array backed by a grounded dielectric slab. However, we control the phase of each unit by varying its size instead of using phase shifters or other conventional methods. Figure 10 shows the simulated radiation patterns of a Hertzian dipole array arranged as shown in Fig. 3(a). In the simulation, the excitation phase is set to ${e}^{-i{\varnothing }_{n}}$, and the array is placed on the FR4 slab backed by a metallic ground. Figure 10 clearly shows that LHCP broadside radiation with small cross-polarization is achieved at 10 GHz.

## Discussions

A novel approach that uses the phase-tuning mechanism for designing broadband MAs is proposed in this study. The MA is structured by assigning RBUs with different sizes, which can introduce phase changes, on a grounded slab. If we make the phase of the currents induced by surface waves equal to ${e}^{\pm i{\varnothing }_{n}}$ by adjusting the size of the RBUs, then a circular polarized antenna can be achieved. In accordance with this method, LHCP and RHCP antennas are designed and fabricated. The measured results show that the proposed antennas can successfully achieve circular polarized broadside radiation with an axial ratio of less than 3 dB in a wide fractional bandwidth of 30%. In addition, the cross-polar level is less than −35 dB compared with the co-polar components at the main lobe directions. The gain of the proposed MAs can be larger than 15.57 dBi for the LHCP and RHCP antennas at 10 GHz. Simultaneously, the 3-dB gain bandwidths of the RHCP and LHCP antennas reach 17% and 22%, respectively, which are considerably larger than those of other MAs reported in recent years. To the best knowledge of the authors, this approach is the first to realize a circularly polarized MA by using the phase-tuning function. The proposed phase-tuning MAs exhibit great potential for satellite and communication systems due to their simple design and satisfactory performance.

## Methods

Numerical simulations are performed using the FEM-based commercial software HFSS. The experimental structure is fabricated using a 3.2 mm thin dielectric film with a dielectric constant of 4.5 and a tangent loss of 0.02. A coaxial probe is used to feed the antenna. We use Agilent’s Vector Network Analyzer (PNA-X5242A) to measure the S parameters (i.e., the reflection coefficient S_11_) and radiation patterns of the fabricated sample. Radiation patterns and antenna gains are measured in a microwave anechoic chamber.
